# The hyper-reactive malarial splenomegaly: a systematic review of the literature

**DOI:** 10.1186/s12936-015-0694-3

**Published:** 2015-04-29

**Authors:** Stefania Leoni, Dora Buonfrate, Andrea Angheben, Federico Gobbi, Zeno Bisoffi

**Affiliations:** Centre for Tropical Diseases, S Cuore Hospital, 37024 Negrar Verona, Italy; Internal Medicine Department, Verona University, Piazzale L A Scuro, 10, 37134 Verona, Italy

**Keywords:** Hyper-reactive, Malarial, Splenomegaly, Management, Treatment, Epidemiology, Review

## Abstract

**Background:**

The hyper-reactive malarial splenomegaly syndrome (HMS) is a leading cause of massive splenomegaly in malaria-endemic countries. HMS is caused by a chronic antigenic stimulation derived from the malaria parasite. Classic Fakunle’s major criteria for case definition are: persistent gross splenomegaly, elevated anti-malarial antibodies, IgM titre >2 SD above the local mean value and favourable response to long-term malaria prophylaxis. The syndrome is fatal if left untreated. The aim of this study is to systematically review the literature about HMS, particularly focussing on case definition, epidemiology and management.

**Methods:**

The search strategy was based on the following database sources: Pubmed, EmBase, Scopus. Search was done in March, 2014 and limited to English, Spanish, Italian, French, and Portuguese.

**Results:**

Papers detected were 149, of which 89 were included. Splenomegaly was variably defined and the criterion of increased IgM was not always respected. The highest prevalence was reported in Papua New Guinea (up to 80%). In different African countries, 31 to 76% of all splenomegalies were caused by HMS. Fatality rate reached 36% in three years. The most frequent anti-malarial treatments administered were weekly chloroquine or daily proguanil from a minimum of one month to lifelong. In non-endemic countries, a few authors opted for a single, short anti-malarial treatment. All treated patients with no further exposure improved. Cases not completely fulfilling Fakunle’s criteria and therefore untreated, subsequently evolved into HMS. It seems thus appropriate to treat incomplete or ‘early’ HMS, too.

**Conclusions:**

For patients not re-exposed to endemic areas, a short course of treatment is sufficient, showing that eradicating the infection is sufficient to cure HMS. Longer (probably lifelong) courses, or intermittent treatments, are required for those who remain exposed. Splenectomy, associated with high mortality, should be strictly limited to cases not responding to medical treatment.

## Background

Hyper-reactive malarial splenomegaly (HMS) represents one of the leading causes of massive splenomegaly in malaria-endemic countries [1]. HMS is caused by an aberrant immune response to a chronic antigenic stimulation in subjects long exposed to malaria parasites [2]. Previously defined as tropical splenomegaly syndrome (TSS), HMS has long been considered distinct from a splenomegaly directly resulting from malarial parasitaemia. The syndrome is characterized by macroglobulinaemia with overproduction of immunoglobulin, especially of the IgM class, which aggregate into high molecular immune complexes and cause persistent splenomegaly because of prolonged clearance from the reticuloendothelial tissue [3]. Cryoglobulins and autoantibodies, such as, for instance, rheumatoid factor, contribute to the macroglobulinaemia [4]. A direct correlation between the spleen size and the IgM titre has been described [5-8]. Genetic factors are likely to be involved in the development of HMS. Studies carried out in Papua New Guinea reported a higher incidence in individuals with HLA-DR2 haplotype or with HLA heterozygosity [9,10]. Moreover a retrospective study carried out in Ghana evidenced that the relatives of patients with HMS were more likely to have splenomegaly than population controls [11].

Diagnostic criteria for HMS were proposed by Fakunle in 1981 [12]. Major criteria are: persistent gross splenomegaly extending more than 10 cm below the costal margin, without any other apparent cause, elevated anti-malarial antibodies, IgM titre >2 standard deviations (SD) above the local mean value and favourable clinical and immunological response to long-term malaria prophylaxis [13]. Minor diagnostic criteria are: hepatic sinusoidal lymphocytosis, normal cellular and humoral immune responses to antigenic challenge, including phytohaemagglutinin stimulation (PHA), hypersplenism, lymphocyte proliferation, occurrence within families or tribes.

Other laboratory findings are related to hypersplenism, such as variable degrees of pancytopaenia, especially anaemia. The underlying mechanism causing anaemia is the plasma volume expansion and the spleen sequestration along with an increased haemolysis. Reticulocytes and indirect bilirubin are often increased [14,15]. Acute episodes of haemolysis can also occur and seem to be associated with an autoimmune, cold-agglutinin-mediated response triggered by non-patent parasitaemia [16]. Sinusoidal lymphocytosis may be revealed by histologic examination of liver biopsy, but it is not specific. The peripheral blood smear in HMS subjects is most often negative. However, using more sensitive diagnostic methods, such as polymerase chain reaction (PCR), the proportion of positive cases increases [17-19].

The main, severe complications of HMS are acute infectious illnesses, haemolytic anaemia (especially during pregnancy) and splenic rupture. According to historical data, the syndrome is often fatal if left untreated [20].

Some authors have hypothesized that HMS could be considered as a pre-malignant state that could evolve to chronic lymphocytic or hairy cell leukaemia or splenic lymphoma with villous lymphocytes, as a result of a multi-step process with a single clone selection in a set of deregulated polyclonal expansion of lymphocytes [21-23]. Actually, HMS and lymphoproliferative disorders are often clinically indistinguishable. Serological similarities between HMS and splenic lymphoma with villous lymphocytes (SLVL) have also been reported. In the latter condition, too, a markedly raised anti-malarial antibody and IgM level have been observed [24]. Spleen reduction after a prolonged anti-malarial treatment is one of the main criteria used to diagnose HMS. Failure to respond makes an alternative diagnosis more likely [25].

Despite a comparatively large amount of literature on malaria, only a few papers deal with this particular complication. Moreover, diagnostic criteria are not uniform, epidemiological data are scarce and the clinical management has been variable. This aim of this review was to retrieve the relevant literature on HMS, focusing in particular on three key aspects: diagnostic criteria for the case definition, epidemiology (prevalence and mortality) and management.

## Methods

### Searching

The search strategy was based on the following database sources: Pubmed, EmBase, Scopus, with no limitation as for the year of publication. The oldest paper retrieved was published in 1966. Our searching strategy was based on these keywords (using of course the specific language of each database): asymptomatic malaria, chronic malaria, malarious splenomegaly, splenomegaly syndrome, splenomegalic syndrome, tropical splenomegaly, malarial splenomegaly. Additional search terms used were: epidemiology, prevalence, incidence, pathogenesis, diagnosis, case definition, prognosis, mortality, fatality, management, treatment.

The search was restricted to ‘humans’ and to the following languages: English, French, Spanish, Italian, and Portuguese. The search was carried out in March 2014. Reference lists of all the articles identified were also examined and relevant cited references were reviewed similarly.

### Types of study

Surveys, epidemiological studies, case reports/series, studies on etiology, pathogenesis, diagnosis and treatment of HMS were collected. Reviews were excluded from the analysis.

### Inclusion criteria

Articles had to deal with hyperactive malaria splenomegaly or its synonymous, such as tropical splenomegaly or chronic malaria, as a main subject.

### Exclusion criteria

Papers were excluded when primarily dealing with acute malaria or with other causes of splenomegaly (such as EBV, CMV, HIV, schistosomiasis). All papers identified were assessed by two reviewers (SL and DB) who independently screened the titles and abstracts, using the criteria mentioned above. Then they read the full text of those retained after the first screening. In case of differences, a final consensus was reached after discussion that included a third reviewer (ZB).

Data were extracted from all finally retained papers and specific fields of an Excel spreadsheet were populated. The spreadsheet was previously created on the basis of the study’s purpose. With available data the analysis was focused first on epidemiology, such as gender and age of patients, country where the study was conducted and country of origin of the patients, prevalence of the syndrome in a given area, mortality rate, etc. Then we filled the section on diagnostic criteria and in particular if they followed the Fakunle’s criteria or not. Finally data were extracted considering the type and duration of the treatment and when available, also data on outcome at follow-up. Finally, the reported treatment outcome of studies with more than 10 recruited subjects was summarized. The studies were obviously heterogeneous but, because of the extreme poverty of papers dealing with HMS, it was aimed to provide the most comprehensive picture of the main reported aspects of the syndrome since the first publication until now.

## Results

The electronic search identified 331 papers from Pubmed, 60 From EmBase and 75 from Scopus. Sixty-eight duplicates were discarded. After an evaluation of language, titles and abstracts, a total of 152 papers were detected that may relate to this review. Thirteen were not retrieved. On the basis of the full text, 51 papers were discarded since they did not deal with HMS.

**Figure 1:**
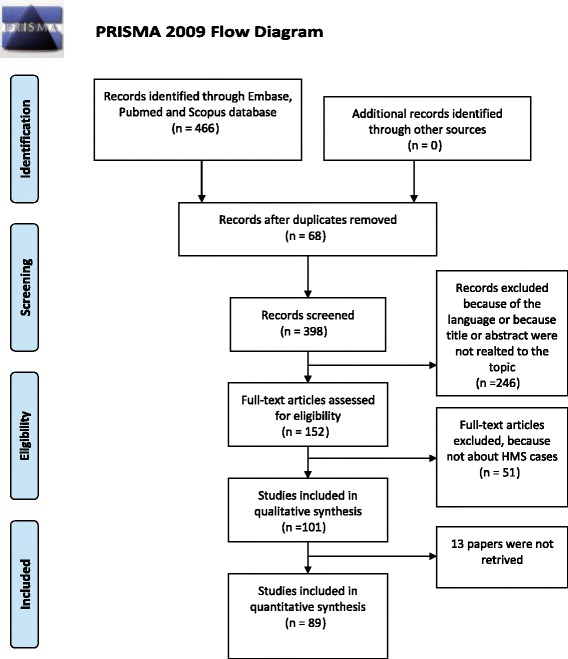
PRISMA flow chart.

The remaining 89 papers were included. The flow is summarized in Figure 1 (PRISMA flow chart).

Sixty-three papers described cases observed or studies conducted in endemic areas. In particular, 50/63 (80%) in Africa, 8/63 (12%) in Asia [26-33], and 5/63 (8%) in South America [16,34-37]. Of the 26 papers from non-endemic countries, 22 (85%) concerned patients observed in Europe, the others were case reports from Israel [38], the USA [39,40] and Hong Kong [41]. Apart from two retrospective studies [42,43] and one prospective longitudinal study [19], the other papers were case reports/case series. These included immigrants (15 papers), expatriates (ten papers) or both groups (one paper). The range of age of subjects under study fell from birth [44] to a maximum of 77 years [45] and most of the patients with HMS were young adults. According to some studies a male gender prevalence was noted but analysing the literature no significant gender difference was observed.

### Diagnostic criteria for case definition

**Figure 2:**
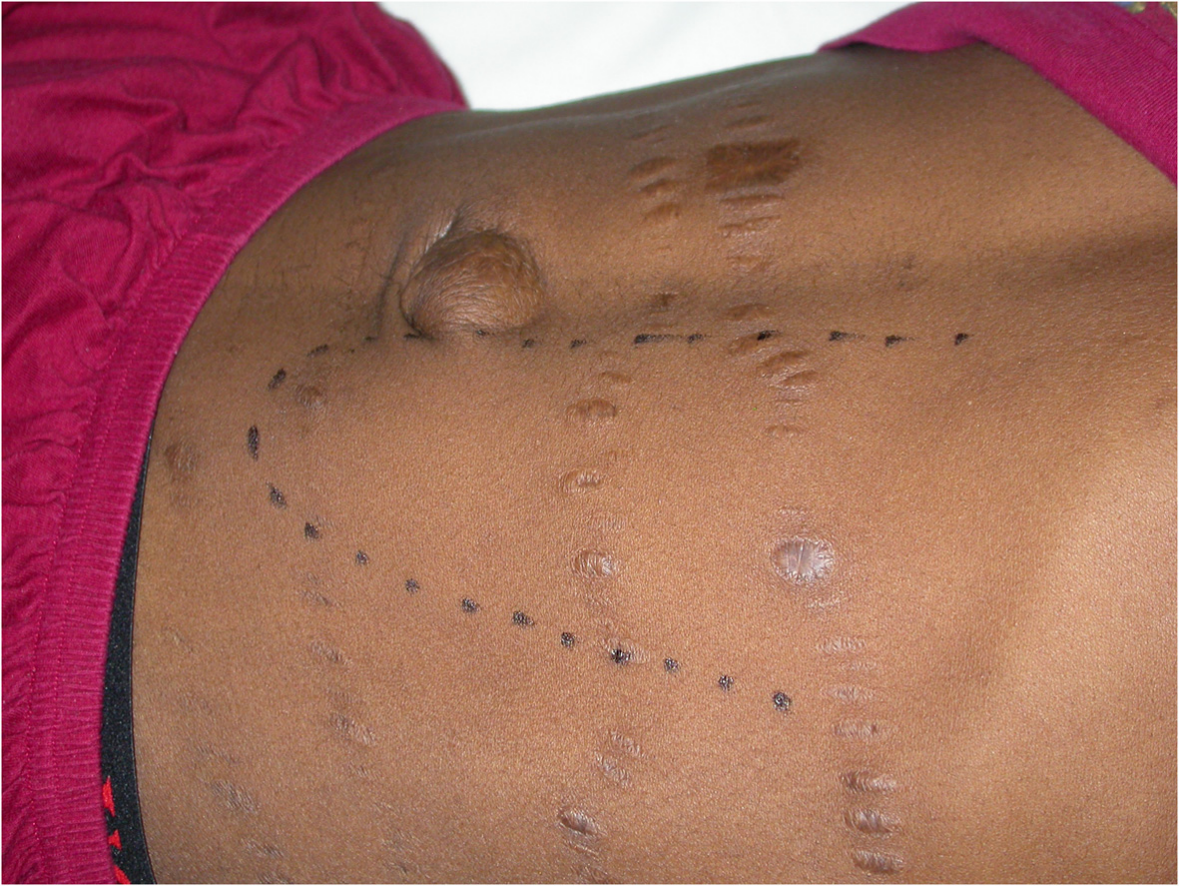
African patient with splenomegaly seen at the Centre for Tropical Diseases, Negrar.

Fakunle’s criteria were mostly used, but adherence to them was variable. Most authors declaring to follow Fakunle’s criteria considered as splenomegalic a patient with spleen size bigger than 10 cm below the costal margin (Figure 2), but lower measures were also reported [34,46]. Overall, the range of the spleen size reported to define splenomegaly was extremely variable, from 3 [27] to 30 cm [47] below the costal margin. Both palpatory and echographic methods were used. Some authors preferred to use Hackett’s spleen size classification [16,20,29,35,48-50], which is based on palpation. They considered as splenomegalic a patient with spleen size classified at least in the II or III grade (Table 1). Other authors did not define splenomegaly, but described the spleen as “gross” [13] or “huge” [39]. The increase in IgM titre was not always respected. Some authors considered it neither specific nor necessary [51,52]. In five studies the syndrome was also diagnosed in absence of raised IgM [1,32,37,52,53].

**Table 1:** **Hackett’s spleen size classification**

**Spleen grade**	**Description of the spleen grade**
0.	Spleen not palpable even on deep inspiration.
I.	Spleen palpable below costal margin, usually on deep inspiration.
II.	Spleen palpable, but not beyond a horizontal line halfway between the costal margin and umbilicus, measured in a line dropped vertically from the left nipple.
III.	Spleen palpable more than halfway to umbilicus, but not below a line horizontally running through it.
IV.	Palpable below umbilicus but not below a horizontal line halfway between umbilicus and pubic symphysis.
V.	Extending lower than class 4.

In other studies the case definition relied on at least two out of the three major criteria (leaving aside anti-malarial antibody titre that is invariably raised), besides excluding other causes of splenomegaly [28,42,43,54]. It was observed that some of those patients, if further exposed to malaria, evolved to the complete syndrome, and this suggested the possibility to make the diagnosis at an early stage [43]. Moreover, a direct correlation between the splenomegaly class and the IgM level has been demonstrated, suggesting a continuum in the syndrome evolution [42].

Another approach to the diagnosis of HMS was a combination of major and minor criteria. In 1976, before the publication of Fakunle’s criteria, Mardsen and Crane had suggested the following: persistent splenomegaly, hepatic sinusoidal lymphocytosis, disproportionate elevation of serum IgM levels and high anti-malarial antibody titre, without considering the response to the therapy [55]. In other papers, the exclusion of other causes of splenomegaly in an endemic area was sufficient to consider a patient as affected by HMS [56,57].

### Epidemiology in endemic countries

There are scanty data regarding the prevalence of the syndrome in the general population. Studies performed in Papua New Guinea reported an extraordinarily high prevalence of the syndrome (85% in five years old children and 80% in adults) among inhabitants of the Upper Watut Valley [58]. The relationship between the parasite rate and the spleen size varied with age: in children the spleen size appeared to be directly related to the parasite rate, while in adults the opposite occurred [44].

Studies conducted in The Gambia reported a much lower prevalence: 1.6 per 1,000 subjects aged ten years or older [44,59]. Other authors studied the proportion of splenomegaly caused by HMS. In northern Nigeria, 30 splenomegalic patients out of 75 (40%) were classified as having HMS, and so were 137 out of 334 subjects in northern Zambia (again, 40%), 38 patients out of 131 (31%) in Kenya, 91/221 (41%) in Ghana and 87/114 (76%) in eastern Sudan [1,48,60-62].

In Papua New Guinea, the overall mortality rate in a series of 75 untreated patients during a 72-month period was 36%, reaching 57% in patients with grade V splenomegaly [20]. In another study, the reported mortality rate in adults was 26% at ten years follow-up, but it fell to 13% in subjects under chloroquine prophylaxis [2]. The main causes of death were infectious diseases, acute haemolysis and multi-organ failure (MOF) [35,63,64].

### Non-endemic countries

The first case of HMS in a Caucasian who lived in Africa was described in 1970 [65]. Only two case series were retrieved describing more than ten expatriates returning to Europe [42,43]. Most patients had lived for at least five years in an endemic area. Both series (in Belgium and Italy, respectively) included patients fully responding to the classic criteria (17/49 and 29/57, respectively) and patients with an incomplete syndrome. The clinical outcome at follow-up was favourable for all patients who remained in Europe and presented at follow-up. The only other case series described in Europe is a prospective study in Spain [19] on 14 immigrants (from Equatorial Guinea or Cameroon), all responded to Fakunle’s criteria. Four had a positive blood film, and four additional patients were positive at PCR.

A six years old child was the youngest immigrant from Africa diagnosed with HMS [66]. A seven years old child, born in UK to Ghanaian parents, was diagnosed with HMS after three short trips to Ghana (eight weeks in total), the last one eight months before presentation. Despite the absence of fever or fever history, *Plasmodium falciparum* trophozoites and gametocytes were found in her blood [67].

### Therapy in endemic countries

The most frequent anti-malarial treatments administered in endemic countries were weekly chloroquine [2,26,31-34,37,49,61,62,68-71] or daily proguanil [13,25,52,57,60,64,71-78]. Other regimens were chloroquine plus primaquine [27,70], mefloquine [28], quinine [18], pyrimethamine [56], artemether [18], and sulphadoxine/pyrimethamine [18,50]. Overall, the duration of therapy ranged from a minimum of one month [13,56] to a lifelong treatment [2]. The largest cohort of patients (148) under lifelong chloroquine prophylaxis showed an improvement of all, with a partial regression of splenomegaly and an increase of haemoglobin level over a period of 12-18 months. However, in no case a normalization of the spleen size was observed. The prophylactic regimen halved the mortality rate over ten years compared to untreated patients [79]. Other studies confirmed the efficacy of this regimen [27,31,71]. The most recent one was carried out in Sudan in 2013 and reported, after a three-month therapy, a complete normalization of the spleen size in 14 patients out of 21 [69]. In Nigeria, 10/39 subjects fully recovered and 29/39 improved after daily proguanil administered for two to 12 months [76].

Two papers reported improvement after a single, short course of anti-malarial treatment followed by oral steroid therapy [50,80]. However, other authors observed that the syndrome tended to quickly relapse once anti-malarial therapy was stopped [75,78]. Only one study in Sudan [18] opted (for the 33 patients included) for a short course anti-malarial treatment (various regimens) as for an acute falciparum malaria. For most of the patients, other treatments were administered during the follow-up (for 15 to 24 months). However, no prophylaxis was administered during the different treatments. Thirty-six patients had improved and 12 had worsened at the end of the follow-up period, while six were reported as unchanged. Table 2 summarizes the main results of the studies including more than ten patients.

**Table 2:** **Treatment outcome: studies conducted in malaria endemic countries with >10 patients and follow up data available**

**Study**	**Country**	**N patients**	**Type of treatment**	**Duration**	**Follow up**	**Outcome**
**Pryor 1967** [49]	*New Guinea*	99	CLQ 1500 mg/3 days, then 300 mg/wk	NR	average 4.1 mths in hospital plus 6-23 mths out	No change in spleen size, general benefit
**Sagoe 1970** [57]	*Nigeria*	43	PG 100 mg/day	≥6 mths	6 mths	32 improved 11 worsened
**Bagshawe 1970** [13]	*Kenya*	28	PG 100 mg/day	1 - 26 mths	1 -6 mths	16 improved 11 worsened 8 unchanged
**Stuvier et al. 1971** [27]	*India*	14	PQ 15 days, then CLQ 300 mg/wk	6-14 mths	once/mths for ≥6 mths	11/14 spleen size ↓50%
**Stuvier et al. 1974** [71]	*Uganda*	41	CLQ 300 mg/wk or PG 100 mg/day	NR	4- 20 mths	all improved
**Bryceson et al. 1976** [60]	*North Nigeria*	30	PG 100 mg/day	3 - 12 mths	3 mths	12/13 improved (17 lost)
**Fakunle and Greenwood 1980** [64]	Nigeria	69	PG 100 mg/day	3 mths	10 wks	2 /40 died plus 8/29 defaulters
**De Cock et al. 1986** [52]	Kenya	38	PG 100 mg/day or CLQ 300 mg/wk	NR	NR	13/18 improved (20 lost)
**Crane 1986** [2]	Papua NG	148	CLQ 300 mg/wk	lifelong	12-18 mths	146 improved (2 lost)
**Gupta et al. 1987** [31]	India	54	CLQ 300 mg, 1 or 2/wk	2 yrs	2 yrs	54 Improved
**Mac Onuigbo and Mbah 1992** [76]	Nigeria	39	PG 100 or 200 mg	2 - 12 mths	2 - 12 mths	All improved (10 cured)
**Manenti et al. 1994** [79]	Tanzania	312	PMT 25 mg/wk	1 mths	3 mths	208 improved 104 unchanged
**A-Elgayoum et al. 2011** [18]	Sudan	54	Single short term treatment (various regimens)	1 d to 1 wk (often repeated)	15 –24 mths	36 improved 12 worsened 6 unchanged
**Alkadarow et al. 2013** [69]	Sudan	33	CLQ 300 mg/wk	3 mths	3 mths	14/21 improved (12 lost)

(*CLQ* = chloroquine; *PG* = proguanil; *PQ* = primaquine; *PMT* = pyrimethamine; *NR* = not reported).

Only few data are available on HMS treatment in pregnancy. A recent retrospective study carried out in Thailand reported the efficacy of weekly mefloquine, administered for two to 25 weeks (median nine weeks) to 31 pregnant women with suspected HMS, without any major adverse event. Those presenting only one or two major criteria of the conventional definition of HMS were also treated and showed on average a spleen size reduction of more than 40% [28].

### Therapy in non-endemic countries

The management of HMS in non-endemic areas has been heterogeneous. Overall, the drugs used were: chloroquine, quinine plus clindamycin or doxycycline or pyrimethamine-sulphadoxine, proguanil, mefloquine, atovaquone-proguanil, halofantrine, and artemisinin derivatives. A few authors preferred to prescribe a short course (≤seven days) of anti-malarial treatment, while the majority opted for longer therapy. Overall, the follow-up period ranged from six weeks to 36 months. In six studies out of 21 a short course of treatment (as for an acute malaria episode) was administered [17,42,43,66,81,82]. In the Belgian study (Table 3) [42] all 49 expatriates received a short course of anti-malarial therapy. Thirty-nine patients were followed up for at least two weeks after treatment (median six weeks) showing improvement or a complete recovery. The Italian study (Table 3) [43] (57 patients, mostly European Caucasians, of which 28 with an incomplete ‘early’ stage HMS) confirmed the efficacy of short treatment in absence of re-exposure, and showed 100% improvement at follow-up (six to 36 months after therapy). The remaining four papers are case reports. Two of them reported a global improvement at follow-up visits, two and 24 months after treatment, respectively [17,82]: one used quinine plus doxycycline and the other one mefloquine. Conversely, one case was described with no improvement eight months after a three-day course of atovaquone-proguanil [46].

**Table 3:** **Treatment outcome: studies conducted in non endemic countries with >10 patients and follow up data available**

**Study**	**Country**	**N patients**	**Treatment**	**Duration**	**Follow up**	**Outcome**
**Van den Ende et al. 2000** [42]	Belgium	39 Caucasians, 17 fully responding to Fakunle criteria	Single, short term treat for Pf malaria (various regimens)	1 day to 1 week according to drug	≥2 wks (average 6 wks)	All not re-exposed improved or fully recovered
**Eseme et al. 2004** [43]	Italy	49 Caucasians (29 fully responding to Fakunle criteria) plus 8 Africans (different countries)	Single, short term treatment for *falciparum malaria* (various regimens)	1- 4 days according to drug	6-36 mths	All not re-exposed improved or fully recovered
**Puente et al. 2001** [83]	Spain	14 Africans (13 from E. Guinea, 1 from Cameroon)	Quinine standard dose for 1 wk then CLQ	3- 9 mths (mean = 4.3 mths)	3 - 9 mths (mean = 4.3 mths)	All not re-exposed improved or fully recovered

The prospective study in Spain (Table 3) [19], that was conducted on 14 African subjects, besides using a short course of anti-malarial treatment just as in the Belgian and Italian studies referred to above [42,43], also added weekly chloroquine for a longer period, ranging from three to nine months, basically obtaining the same clinical results (in those not re-exposed) as in the two previous studies. Most other authors opted for long-term treatment following, in some cases only, a short anti-malarial course [84,85]. The duration of therapy varied from one [46] to 12 months or until recovery [83,86]. Intermittent therapy was also used, with quinine [45] or with chloroquine plus proguanil [87].

The most common prescription, just as in endemic countries, was chloroquine alone [54,83,85,88], or in combination with doxycycline [46], with proguanil [42,85,89] or with primaquine [90], or preceded by a short course of quinine [19,83,88] or halofantrine [83]. Other regimens used for long-term treatment were proguanil [67,85,86], mefloquine [91] or quinine plus doxycycline [17].

All patients with no further exposure and with available follow-up data improved. It is not clear whether a mere withdrawal of exposure, without treatment, could be sufficient to obtain HMS resolution: only a few case reports debate on this aspect. One described the persistence of HMS in a eight years old Caucasian boy, who had been on chloroquine prophylaxis while in Africa, after an 18-month stay in Europe [91]. Another case of HMS was diagnosed in an Ethiopian immigrant after a 19-month stay in Israel [38]. On the contrary, a spontaneous resolution of the syndrome was reported in a Guinean patient after two months of residence in a malaria-free area, with a regression of the spleen size from 20 cm to 3-4 cm below the costal margin and a normalization of all laboratory findings [92]. The main results of the three case series described in Europe with more than ten patients are summarized in Table 3.

### Splenectomy

Splenectomy in HMS is generally suggested for patients with huge splenomegaly and disabling symptoms, who do not respond to medical treatments [29,93]. One study analysing short- and long-term outcome of splenectomy reported a peri-operative mortality rate of about 31% rising to 39% at long-term (41-74 months) follow-up [94]. The most frequent peri-operative complications described were major bleeding and infections. The latter were the main cause of death in the following months. Most surviving patients improved. However, the liver tended to progressively enlarge, probably related to persistence of the antigenic stimulus [95]. In non-endemic areas, therapeutic splenectomy has been reported in Venezuela, Sri-Lanka, Hong Kong [30,39,41] and the USA (for haemolytic anaemia complicating HMS) [40].

## Discussion

The literature available on HMS consists of some case reports and a few case series. Parameters considered by different authors are often heterogeneous and hardly comparable. However some key points are identifiable. First of all, HMS should be taken into consideration in all patients with splenomegaly living/having lived in malarious areas. Strict adherence to Fakunle’s criteria is questionable. Some authors observed that cases not completely fulfilling the criteria can subsequently evolve into the full-blown, ‘classical’ HMS [42,43], thus representing an early stage of the syndrome. Considering the high fatality rate of this condition, if left untreated, as well as the wide availability of effective anti-malarial drugs, it seems appropriate to treat HMS even in case the classical triad of splenomegaly, raised IgM and anti-malarial antibodies is only partially satisfied. Clearly, investigations of other possible causes of splenomegaly should be carried out, in particular, but not limited to, considering lymphoproliferative disorders.

The treatments administered have been heterogeneous and do not permit recommending a first choice regimen yet, considering the lack of randomized clinical trials. However, for patients who move from endemic to non-endemic areas, there is circumstantial evidence supporting the fact that an adequate short-course treatment is effective [42,43]. This contrasts with the more common choice of a long-term anti-malarial course in these cases, too, which is the classical approach that has been suggested and followed for decades. It reflects the view that the syndrome tends to evolve even though malaria parasites are no longer present in blood. This particular aspect was described in a study showing a progressive enlargement of the liver after splenectomy in a patient with negative thick blood films. This occurrence was attributed to persistence of the underlying stimulus to reticulo-endothelial system [95]. According to the most cited authors [2], the absence of parasitaemia is one of the diagnostic criteria. This view does not hold any longer. On the one hand, recent reports showed that more sensitive diagnostic tools have found a higher proportion of malarial infection in HMS patients [17]. This suggests that *Plasmodium* is present, albeit often at sub-microscopic parasite density. On the other hand, the full response to a short course of anti-malarial therapy indicates that the eradication of the infection is usually sufficient to cure HMS [28,42,43]. Therefore, the presence of *Plasmodium* in blood is necessary for a further development of the syndrome. This is also suggested by the unusual case of HMS reported above [67]: a child who still had circulating *Plasmodium* eight months after her last short-term visit to a malaria-endemic country.

## Conclusion

Lifelong effective malaria prophylaxis or intermittent treatments are probably necessary for those who remain exposed to malaria transmission. Chloroquine seems to still be partially effective, even if *P. falciparum* resistance has developed. Possibly this regimen acts not only as an anti-malarial, but also as an immunomodulating and immunosuppressant therapy [47,96], as it is suggested by the regression of the spleen size even in patients with lymphoproliferative disorders [60]. The choice of the drug should consider the local pattern of *P. falciparum* drug susceptibility, as well as the availability and cost of the different regimens. Splenectomy is potentially associated with high mortality, therefore it should be strictly limited to cases that do not respond to medical treatment [94,97].
